# Supplementary material for: Mitochondrial Haemoglobin Is Upregulated with Hypoxia in Skeletal Muscle and Has a Conserved Interaction with ATP Synthase and Inhibitory Factor 1
Source: Cells. 2023 Mar 16;12(6):912. doi: 10.3390/cells12060912 (PMC10047868; doi:10.3390/cells12060912)
Supplement: Supplementary file 1 [file cells-12-00912-s001.zip › cells-2264602-supplementary.pdf]

Hb ATP supplementary data sheet

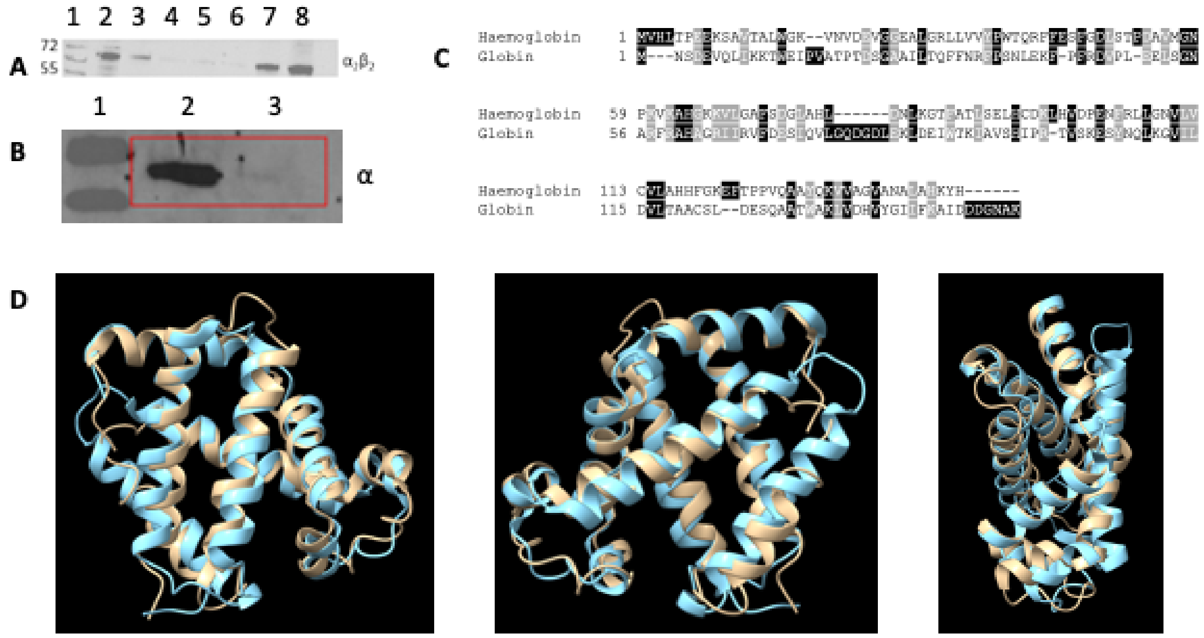

Supplementary Figure S1 *– A) Co-IP of haemoglobin subunits in rat liver, 1 = molecular weight marker, 2 = rat liver lysate, 3 = unbound lysate proteins, 4 = extraction buffer 1, 5 = extraction buffer 2, 6 = extraction buffer 3, 7 = last wash buffer, 8 = elution buffer; B) Co-IP of haemoglobin alpha in Notothenioids with IF1, 1 = molecular weight marker, 2 = unbound lysate protein, 3 = elution buffer; C) Sequence homology of H. sapiens haemoglobin* β *and D. melanogaster globin 1; D) Structural alignment of H. sapiens haemoglobin* β *(brown) and D. melanogaster globin 1 (blue).*

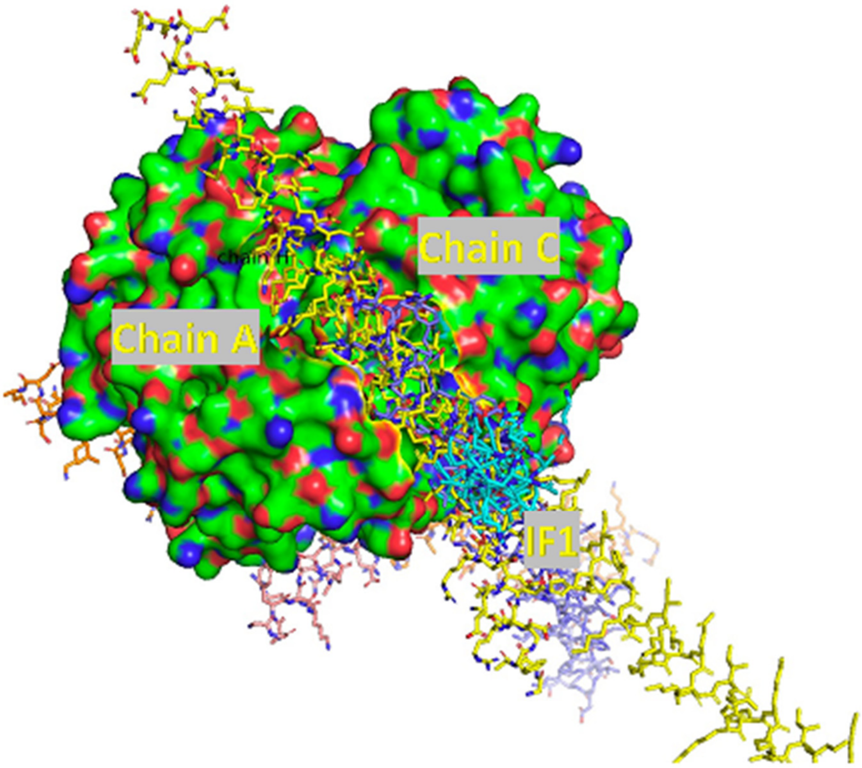

Supplementary Figure S2 *- Top 10 best ranked Docking results for Hb-all chains and IF1. Hb is represented by a molecular green surface, the IF1 structures are represented by different colours.*

Supplementary Table S1 *– IF1 interacting residues with Hb. The first part of the table shows residues interacting between IF1 and HbA, and IF1 and Hb all chains from the FireDock best docking pose. Second half of the table shows stable interacting residues that remained through the MD trajectory.*

Before simulation

**IF1**	**HbA**	**Hb-all chains**		**IF1**
	Chain C	Chain A	Chain C	
Arg35		Asp85		Arg35
Lys39		Asp85, Ser81		Lys39
Leu42		Ser138	Val1	Leu42
Ala43		Gly78, Asp75, Pro77		Ala43
Leu45			Leu73, Asp74, Leu2, Lys7, Val1	Leu45
Lys46		Pro77, Asn131	Val1	Lys46
Lys47		Asp74		Lys47
Lys49			Pro77, Thr134, Asn131, Val135	Lys49
Glu50		Leu2		Glu50
Glu52	Arg92		Asp75, Gly78, Pro77	Glu52
Ile53			Ser138	Ile53
Ser54		Ala4		Ser54
His56	Ala88, Thr137, Ser138		Ser81, Pro77	His56
Ala57				Ala57
Glu59	Pro95			Glu59
Ile60	Thr134, Ser138		His89, Asp85	Ile60
Arg62	Lys99			Arg62
Leu63	Lys99, Thr134, Ser133, Ala130			Leu63
Gln64	Val1			Gln64
Glu66	Lys99			Glu66
Ile67	Ala130, Asn131, Lys127			Ile67
His70	Asp126, Lys127			His70
Lys71	Lys127, Asp6, Ser3, Leu2			Lys71
Ile74	Lys127, Ala123, Asp6			Ile74

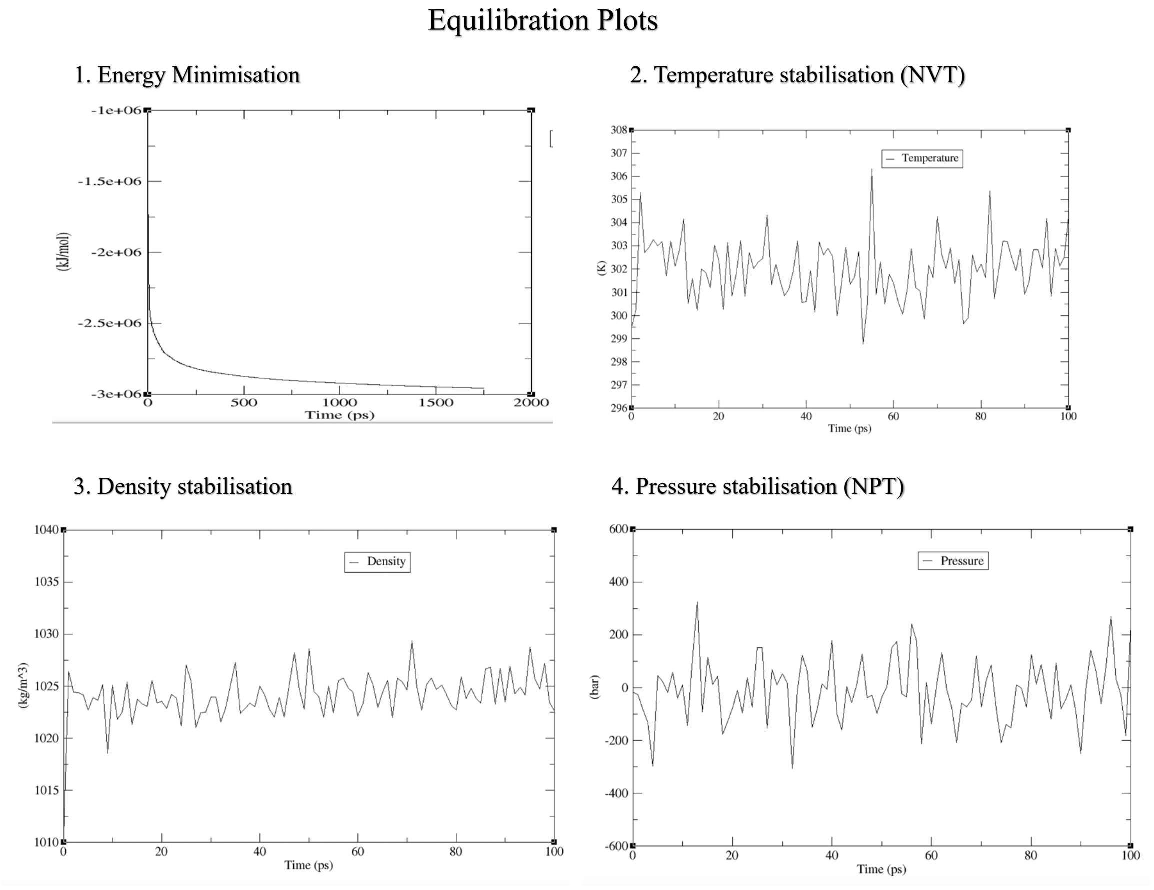

Supplementary Figure S3 *- MD Simulation for Hb and IF1. a) Energy Minimisation of the system, demonstrating a nice steady convergence of potential energy. b) Temperature equilibration (NVT), the plot shows that temperature ranges 302-306 K (average temperature 305 K). c) Pressure equilibration, the pressure fluctuates but in the expected range maintaining the average value. d) Density equilibration, the density plot is very close to the expected values and is stable over time.*

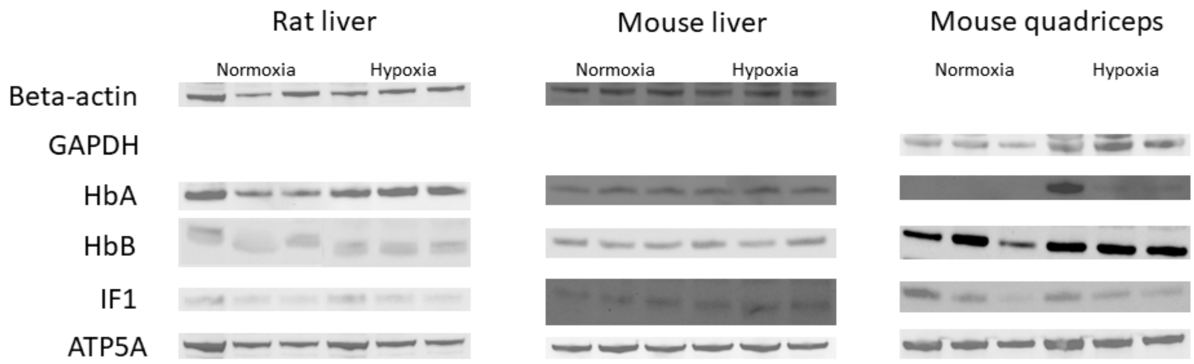

Supplementary Figure S4 *– Western blot relative expression bands for rodent hypoxia tissues (N=3). Beta actin is a loading control in rat liver mitochondria and mouse liver mitochondria samples, GAPDH is a loading control in mouse quadriceps mitochondria samples.*

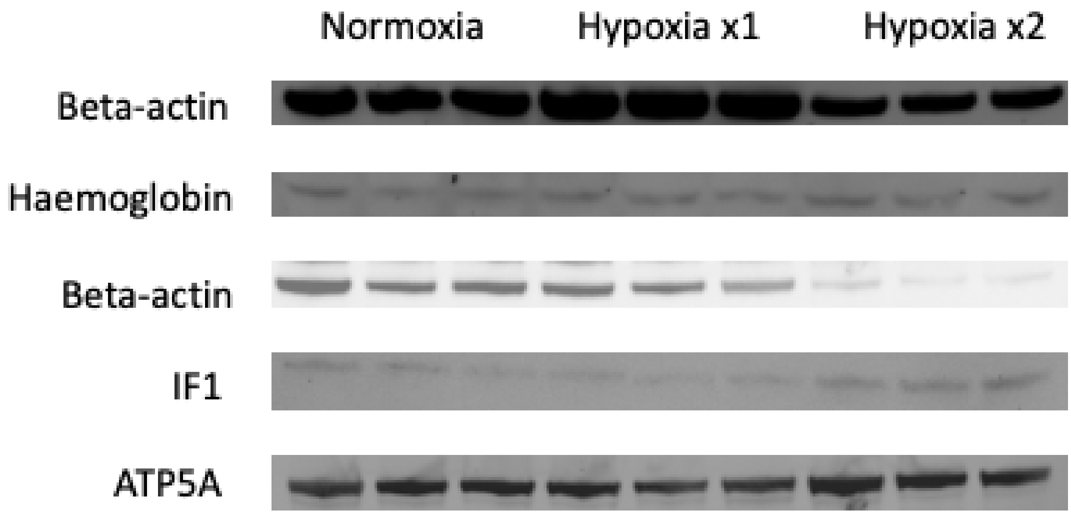

Supplementary Figure S5 *– Western blot relative expression bands for hypoxia exposed D. melanogaster mitochondrial isolates (N=3). Beta actin is a loading control.*

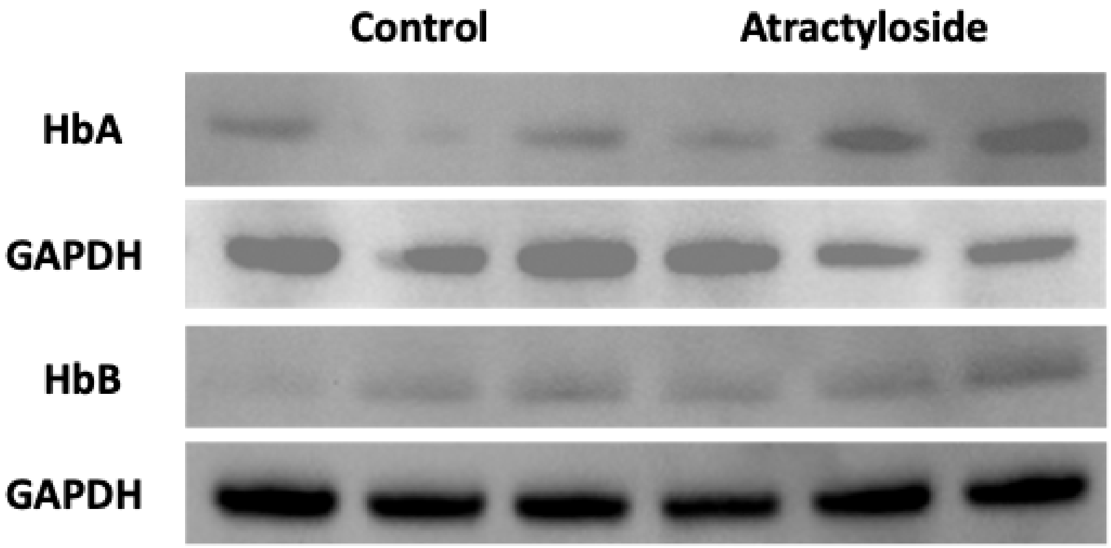

Supplementary Figure S6 – *Western blot relative expression bands for haemoglobin α, haemoglobin β, and GAPDH in mitochondria isolated from control and atractyloside treated HEPG2 cells.*
